# The role of the epithelial sentinels, Langerhans cells and γδT cells, in oral squamous cell carcinoma

**DOI:** 10.1111/prd.12544

**Published:** 2024-01-25

**Authors:** Avi‐Hai Hovav, Asaf Wilensky

**Affiliations:** ^1^ Institute of Biomedical and Oral Research, Faculty of Dental Medicine Hebrew University Jerusalem Israel; ^2^ Department of Periodontology, Hadassah Medical Center, Faculty of Dental Medicine Hebrew University of Jerusalem Jerusalem Israel

**Keywords:** immune sentinels, Langerhans cells, oral epithelium, γδT cells

## Abstract

Oral squamous cell carcinoma (OSCC) arises in the oral epithelium, a tissue in which immune surveillance is mediated by its primary resident leukocytes, Langerhans cells (LCs), and γδT cells. Under steady‐state conditions, LCs and γδT cells play a critical role in maintaining oral mucosal homeostasis. As antigen‐presenting cells of stratified epithelia, LCs respond to various challenges faced by the epithelium, orchestrating innate, and adaptive immune responses in order to resolve them. γδT cells also sense diverse epithelial insults and react rapidly through cytokine production and cytolytic activity. These epithelial sentinels are also considered to be the first leukocytes in the oral epithelium to encounter early carcinogenic events that have the potential of becoming OSCC. As evident in many malignancies, leukocyte populations help prevent cancer development although they also promote tumor progression. OSCC is no exception, as studies have reported both anti‐ and pro‐tumor roles of LCs and γδT cells. In this review, we summarize the ontogeny of LCs and γδT cells in the oral epithelium and discuss their role in OSCC.

## 1 ORAL SQUAMOUS CELL CARCINOMA

Oral squamous cell carcinoma (OSCC) is the most prevalent cancer in the oral cavity, accounting for more than 90% of oral malignancies.^1^ Despite recent advances in the detection, prevention, and treatment of OSCC, this highly aggressive cancer remains associated with a poor 5‐year patient survival rate.^2^ Approximately, one‐third of treated patients experience local or regional recurrence and/or distant metastasis. This poor prognosis can be attributed to the notion that about two‐thirds of patients with OSCC are already at an advanced stage of the disease at the time of diagnosis. The main risk factors for oral cancer are exposure to exogenous carcinogens, such as tobacco smoke, smokeless tobacco, excess alcohol, and the presence of human papillomavirus (HPV).^3^ Nevertheless, it is likely that other risk factors have yet to be identified.

## 2 THE ORAL EPITHELIUM—NOT JUST A PHYSICAL BARRIER

The oral mucosa is constantly exposed to microorganisms, dietary and airborne antigens/substances, that may be harmful and even carcinogenic. Oral carcinoma arises from epithelial tissues exhibiting robust cellular and molecular events, ranging from atypical hyperplasia, which is considered to be the precursor of a carcinoma, to a tumor and, ultimately, to metastatic cancer. To protect against carcinogenesis, the oral mucosa is covered with stratified squamous epithelium that is in a constant process of turnover. The oral mucosa also contains functionally distinct niches such as the gingiva, tongue, buccal, hard palate, and sublingual mucosae, allowing special adaption of the epithelium to the physiological task of each oral niche.^4^ Such niche‐specific alterations in the epithelium are associated with the risk of certain niches containing a higher propensity to cancerous transformation. For example, thin nonkeratinized epithelia, as are found in the floor of the mouth and the ventral surface of the tongue, are at greater risk for OSCC than the gingiva and hard palate.^5^

Besides its elementary physiological protective function, the oral mucosa also harbors a sophisticated immune network.^6^ In fact, the presence of stratified squamous epithelium endows the oral mucosa with mucosa‐ and skin‐like immunological characteristics. Similar to the skin epidermis, the oral epithelium is embedded with two major local sentinels, Langerhans cells (LCs) and γδT cells, providing innate and adaptive immune responses that are responsible for preventing epithelial carcinogenesis.^7^, ^8^ Yet, the epidermis and the oral epithelium vary in their accessibility to leukocytes, as the epidermis is sealed off before birth to circulating leukocytes, whereas the oral epithelium promotes it. This infers fundamental differences in the ontogeny of the LCs and γδT cells in the epidermis versus the oral epithelium, and likely affects their ability to sense and react to prevent epithelial cancerous transformation. This review summarizes the latest research advances on these oral epithelial resident cells and discusses their role in the development and progression of OSCC.

## 3 ORAL LANGERHANS CELLS

LCs are special antigen‐presenting cells (APCs) exclusively located in stratified squamous epithelia such as the skin epidermis and the oral mucosa.^7^, ^9^ Their strategic location highlights their importance as the first line of defense against attacks through the epithelium, such as epithelial carcinogenesis. At steady state, LCs monitor the epithelium and migrate to the lymph node (LN) where they present self or commensal microbial antigens to T cells in order to induce tolerance and maintain tissue homeostasis.^10^ Under inflammatory conditions, such as an epithelial infection or transformation of epithelial cells, the LCs undergo a maturation process accelerating their migration to the lymph nodes (LNs). Within the LNs, LCs activate CD8^+^ and CD4^+^ T cells and polarize their differentiation into various effector cells, a process that is greatly influenced by the nature of the immunological challenge.^11^

**FIGURE 1:**
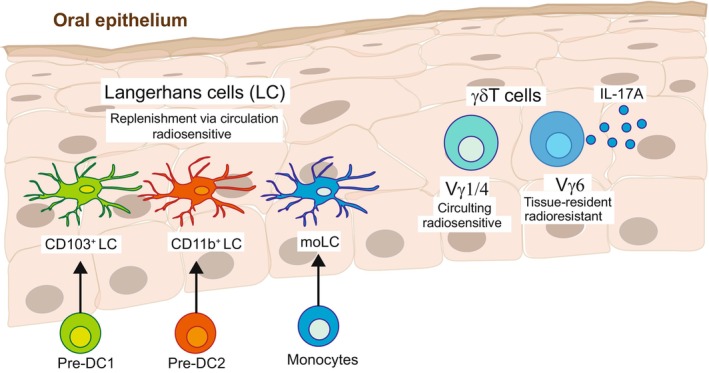
Langerhans cells (LCs) and γδT cells—sentinels of the oral epithelium. The stratified oral epithelium contains two major types of leukocytes, Langerhans cells (LCs) and γδT cells. LCs are antigen‐presenting cells (APCs) that survey the epithelium and respond to a variety of challenges, such as epithelial transformation, by migrating to the lymph node to prime cellular immunity. The epithelium instructs the development of LCs, which arise and continuously replenish from adult bone marrow precursors, pre‐DCs, and monocytes. Pre‐DC1 and Pre‐DC2 give rise to the CD103^+^ LC and CD11b^+^ LC subsets, respectively, while moLCs originate from monocytes. γδT cells are a unique T‐cell subset that is enriched in peripheral epithelial tissues. By rapidly producing cytokines, oral γδT cells contribute to local homeostasis and also to immune surveillance. Oral γδT cells can be segregated into various subsets mainly of Vγ6^+^ and to a lesser extent Vγ4^+^, and Vγ1^+^. The Vγ6^+^ cells are derived from embryonic precursors and are tissue‐resident, self‐renewing, and radioresistant. Vγ4^+^ and Vγ1^+^ cells, on the other hand, are circulating and replaced constitutively and independently of the tissue‐resident population. The Vγ6^+^ subset and also Vγ4^+^ are naturally committed to the rapid production of interleukin‐17A (IL‐17A). Of note, the Vγ5^+^ and Vγ7^+^ subsets are not indicated because their frequencies in adult epithelium are very low. Nevertheless, Vγ5 is of fetal origin while Vγ7 develops after birth.

In addition to their unique anatomical location and their basic APC phenotype based on the expression of CD11c and MHCII, LCs can be distinguished according to the surface expression of the C‐type lectin receptor, langerin (CD207). Langerin is involved in antigen capture, and it induces the formation of Birbeck granules, which are unique rod‐ or tennis racket‐shaped endocytic vesicles considered the hallmark of LCs in mice and humans.^12^, ^13^ LCs also express the epithelial cell adhesion molecule (EpCAM; CD236), a cell‐surface protein that is characteristic of murine epithelia,^14^, ^15^ which also modulates the migration and activation of the LCs.^16^, ^17^ Despite residing in similar anatomical regions and expressing LC‐associated markers, LCs in each type of epithelium vary in their ontogeny.^7^, ^18^ Skin LCs arise from embryonic precursors that seed the developing epidermis before birth and differentiate into LCs immediately after birth.^19^ The differentiated LCs then form a relatively homogenous and radioresistant cellular network that maintains itself locally by self‐renewal throughout life.^20^, ^21^ In contrast, oral LCs develop after birth and are continuously replenished from circulating bone marrow (BM)‐derived predendritic cells (pre‐DCs) and to a lesser extent, monocytes (Figure 1).^22^, ^23^, ^24^ Upon entering the lamina propria, LC precursors are exposed to BMP7, a member of the TGF‐β1 superfamily, that directs their translocation to the oral epithelium where upon local TGF‐β1/ALK5 signaling drives their differentiation into LCs.^25^ Whereas skin LCs are considered a homogenous population, oral LCs can be further divided into at least three subsets: LC1 (CD11b^low^CD103^+^), LC2 (CD11b^+^CD103^−^), and monocyte‐derived LCs (CD11b^+^CD64^+^). In humans, skin epidermal LCs develop from an embryonic origin^26^ and can be identified by the expression of MHCII, CD11c, langerin, and CD1a.^27^ Human oral LCs also exhibit a higher expression of MHCII as well as the costimulatory molecules CD40, CD80/B7.1, and CD86/B7.2.^28^, ^29^, ^30^ Some oral LCs also express CD64 and CD16,^28^ suggesting that, similar to mice, part of these cells arise from monocytes. Another essential difference between skin and oral LCs is the influence of the microbiota on their development. While the differentiation and maintenance of skin LCs are unaltered in germ‐free mice, the frequency and morphology of oral LCs are impaired in these mice.^25^, ^31^ Despite ontogenetic disparities between epidermal and oral LCs, they share many similar transcriptomic signatures and immunological functions, suggesting that LCs from both sources can arise from various precursors in a tissue‐dependent manner.^18^, ^25^

## 4 THE ROLE OF ORAL LANGERHANS CELLS IN OSCC

Due to their epithelial location, LCs are thought to be the first APCs to encounter and react to early carcinogenic events in the epithelium. This is supported by evidence that known risk factors of OSCC, such as tobacco use (including cigarette smoking), alcohol consumption, aging, and HPV infection,^32^, ^33^, ^34^, ^35^, ^36^, ^37^, ^38^ are involved in the disruption of oral LC development and function. Yet previous studies have generated contradicting evidence for the role of epidermal and oral LCs during the development of skin and oral SCCs. In mice, epidermal LCs were reported to have anti‐tumor activity in a carcinogen‐induced skin SCC,^39^, ^40^ whereas other studies found a deleterious impact of LCs in this disease.^41^, ^42^ The role of oral LCs in OSCC also remains vague since observations in humans suggest both anti‐ and pro‐tumor roles for these cells. Several studies have demonstrated reduced numbers of oral LCs in human OSCC,^33^, ^43^, ^44^ while elevated numbers compared to normal tissues were reported by others.^45^, ^46^ In oral epithelial dysplasia (OED), an oral pathology with malignant potential, the numbers of LCs increase as the severity of the OED lesions increases but were significantly reduced in lesions with malignant transformation.^47^ The numbers of LCs in OSCC have been reported as being either increased^48^ or reduced^49^ as compared to OED. Thus, it is difficult to draw conclusions about the role of oral LCs in OSCC based on clinical observations, highlighting the need for an experimental approach to gain insight into this important topic.

**FIGURE 2:**
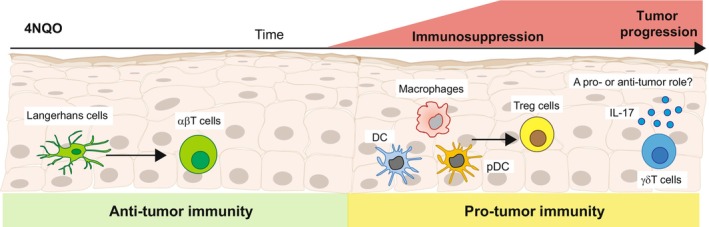
The role of LCs and γδT cells during experimental OSCC. Exposure of mice to the carcinogen 4NQO in drinking water induces rapid migration of LCs to the lymph node where they prime protective CD4^+^ and CD8^+^ αβT cells. The carcinogen, however, dysregulates the repopulation of epithelial APCs, and instead of LCs, macrophages, DCs, and pDCs developed locally. The APCs exert immunosuppressive conditions in the epithelium, in part, by generating a large population of T regulatory (Treg) cells, facilitating the establishment and progression of the tumor. In this model, γδT cells appear to have no impact on tumor establishment, however, due to their capacity to produce IL‐17A they might play a deleterious role in later stages, an issue requiring further investigation.

Using a murine model of OSCC induced by 4‐nitroquinoline 1‐oxide (4NQO), a carcinogen that mimics the various stages of the disease in humans,^50^ LCs were reported to inhibit cancer development.^51^ Carcinogen‐mediated DNA damage in epithelial cells facilitated LC migration to the LN which, in turn, primed CD4^+^ and CD8^+^ T cells, leading to the elimination of damaged epithelial cells. In contrast to carcinogen‐induced epidermal SCC, in which LCs mediated anti‐tumor activity by recruiting natural killer (NK) cells,^40^ NK cells were not recruited by LCs to the oral epithelium.^51^ The carcinogen, however, rapidly altered the APCs residing in the oral epithelium, and APCs other than LCs, such as macrophages, DCs, and plasmacytoid DCs, developed locally. The newly differentiated APCs displayed an immunosuppressive phenotype, facilitating the development of a large population of CD4^+^ T regulatory cells that promoted the establishment of the tumor (Figure 2).^51^ Interestingly, metabolic changes induced in the oral epithelium by the carcinogen, particularly the reduction in oxidative phosphorylation signaling, were proposed to dysregulate the differentiation of the LCs.^51^ This points to a protective role for LCs in the early stages of OSCC, which is in line with observations of reduced LC numbers in premalignant lesions. Since LC differentiation is instructed by signals provided locally by the epithelium, the shift in epithelial APCs might represent a transition point in which the oral epithelium turns into a tumor‐permissive environment.

In addition to their early protective activity, the presence of LCs within the tumor was further suggested to reflect a better prognosis for head and neck SCC (HNSCC) patients.^52^, ^53^ This correlation was also described with regard to laryngeal SCC in which LC infiltration is associated with longer disease‐free survival.^54^, ^55^ It is not clear, however, which mechanisms direct the development of LCs in the tumor, and whether pre‐DCs and/or monocytes contribute to the intratumor LC population. TGF‐β, the cytokine governing LC differentiation at steady state^56^ is expressed in OSCC^57^, ^58^ and thus might mediate this process. Notably, TGF‐β signaling in carcinogenesis is complex and can shift from a tumor‐suppressing to a tumor‐promoting cytokine. Moreover, TGF‐β is involved in epithelial‐mesenchymal transition,^59^ a process that also controls LC differentiation and activation.^60^ Thus, it is hard to predict the effect of TGF‐β on the differentiation of intertumoral LCs and their impact on the disease. This cytokine might ultimately play both anti‐ and pro‐oncogenic roles, depending on the stage of cancer progression. In agreement with the early protective role of LCs, TGF‐β was reported to act as a potent tumor suppressor during early carcinogenesis, whereas during advanced OSCC stages, TGF‐β facilitates tumor progression.^61^

## 5 ORAL γδT CELLS

Another type of tissue‐resident leukocytes in the oral epithelium are γδT cells, a T‐cell lineage expressing the γδT‐cell receptor (γδTCR) rather than the αβTCR expressed by conventional T cells (i.e., CD4^+^ and CD8^+^ T cells). γδT cells are mainly positioned in epithelial barrier tissues and are present at very low percentages in the circulation and secondary lymphoid organs. In contrast to αβT cells, γδT cells mostly act in a manner independent of MHC class I‐mediated antigen presentation.^62^, ^63^ In fact, the information regarding the cognate ligands of the γδTCR is incomplete,^64^ and it appears that γδT cells respond to stress‐induced self‐ligands.^65^ However, uncovering precise recognition signals by the γδTCR requires further clarification.

Intraepithelial γδT cells in barrier tissues display an activated phenotype. The different subsets of these γδT cells harbor various immunological capacities such as cytolytic activity, immunoregulatory capabilities, and rapid secretion of inflammatory cytokines upon stimulation.^66^, ^67^ Tissue‐resident γδT cells are thus considered to play an important role in sustaining epithelial integrity and homeostasis.^67^, ^68^ In adult mice, oral γδT cells are composed mainly of cells expressing Vγ6^+^ (~60%) and to a lesser extent Vγ1^+^, Vγ4^+^, Vγ5^+^, and Vγ7^+^ subsets^69^, ^70^ (Figure 1). Some of the oral γδT subsets are naturally committed to the rapid production of interleukin‐17A (IL‐17A), and they seed the oral mucosa, prenatally.^71^ The TCR repertoire of IL‐17A‐producing γδT (γδT17) cells is enriched in a semi‐invariant Vγ6^+^ and Vγ4^+^ TCR.^72^, ^73^ Whereas the Vγ6^+^ cells are tissue‐resident and radioresistant with self‐renewing capability, Vγ4^+^ and Vγ1^+^ cells constitute approximately half of the intraepithelial γδT cells which circulate and are replaced constitutively and independently of the tissue‐resident population. Oral Vγ6^+^ cells, and other γδT17 cells (such as Vγ4^+^ cells), contribute to establishing homeostasis within the local microbiota,^70^, ^74^, ^75^ while the microbiota regulates the frequency and activation state of oral Vγ6^+^ cells.^69^, ^70^ In humans, the Vδ2^+^ subset is the first γδT cells generated prenatally that is almost exclusively paired with a Vγ9^+^ chain.^76^, ^77^ The Vγ9Vδ2^+^ cells are abundant in the circulation and secondary lymphoid organs. The Vδ1^+^ cells are generated after the Vδ2^+^ subset, a few months after birth.^76^ Vδ1^+^ cells account for one‐third of circulating γδT cells, but they are the predominant γδT cell subset in mucosal barriers, contributing to tissue homeostasis.^78^ An additional subset of γδT cells is Vδ3^+^ cells, which are rare in the circulation of healthy individuals but represent a prominent population in the intestine^79^ and liver.^80^

## 6 γδT CELLS AND ORAL CANCERS

Studies in mice and humans demonstrated that γδT cells have both anti‐tumor and pro‐tumor activities.^81^, ^82^, ^83^ These contrasting roles might be related to the notion that murine γδT cells are prone to produce IL‐17A, a cytokine capable of promoting cancer development,^84^, ^85^ whereas human γδT cells have potent cytotoxic capabilities^86^ and are thus likely to play an anti‐tumor role. Regardless, γδT cells are capable of expressing various receptors that sense and clear cells expressing stress signals, thereby containing tumor immunosurveillance capabilities.^67^, ^87^, ^88^, ^89^ In agreement with this notion, ex vivo stimulation of γδT cells, isolated from the peripheral blood of oral cancer patients, with heat shock proteins (HSPs, known ligands for γδT cells) resulted in clonal expansion of Vγ9Vδ2^+^ cells.^90^ This subset is capable of recognizing HSP60 on oral tumor cells^91^ and lysing autologous and allogenic esophageal tumors via recognition of HSP60 and HSP70.^92^ Human Vγ9Vδ2^+^ cells were also shown to efficiently kill OSCC cell lines that express stress‐induced NK2GD ligands.^93^ A bioinformatic analysis further revealed that γδT cells have prognostic value and even treatment potential in patients with HNSCC, as high abundance of intratumoral γδT cells correlates with better prognosis.^94^ Yet, SSCs were reported to recruit γδT cells producing either IL‐17A or IFN‐γ, depending on the tumor stage. While Vδ1^+^ T cells infiltrated SSC tissues, elevated frequencies of infiltrating Vδ2^+^ T cells and Tregs differentially correlated with early and advanced tumor stages, respectively.^95^ This indicates that human γδT cells likely play a protective role in OSCC. However, Vγ6^+^ and Vγ4^+^ γδT17 cells were suggested to have pro‐tumor activity in various cancers.^84^, ^96^, ^97^, ^98^ An immunosuppressive role was also proposed for γδT cells in OSCC, based on their higher proportion in OSCC patients that was not correlated with apoptosis of tumor cells.^99^ In mice, periodontitis‐associated microbiota can activate γδT17 cells, promoting infiltration of M2‐tumor‐associated macrophages and facilitating OSCC.^100^ γδT cells have no impact on the early stage of carcinogen‐induced OSCC in mice.^51^ Since γδT17 cells represent the majority of oral γδT cells, these cells might have a deleterious impact on OSCC progression.

Regardless of their precise role in various cancers, there is renewed interest in utilizing γδT cells for cancer immunotherapy.^101^ γδT cells recognize a wide range of antigens in an MHC‐dependent and ‐independent manner, thus, these cells could act against tumors with low mutational burdens and downregulated MHC expression. The antitumor capabilities of γδT cells encompass activation of αβT cells and NK cells and their genetic structure allows relatively easy manipulation for therapeutic interventions, thus, harnessing γδT cells for cancer immunotherapy is an attractive approach.

## 7 CLINICAL RELEVANCE

The relationship between LCs and immunosurveillance of human OSCC has been examined by many studies, which indicate overall poor surveillance of carcinogenic events due to reduced levels of oral LCs.^44^, ^47^, ^48^, ^49^, ^102^, ^103^, ^104^, ^105^, ^106^ Moreover, the density of LCs has also been correlated with histological grades of OSCC,^44^ and their number has even been suggested to be a strong and independent prognostic factor for OSCC.^53^ However, a better understanding of the ontogeny and function of LCs is essential for designing new or improved LC‐based therapeutic approaches. Regarding γδT cells, OSCC patients have been shown to have a higher proportion of γδT cells compared to healthy controls, with no correlation to tumor stage.^99^ A high abundance of intratumoral γδT cells has also been reported to correlate with better prognosis, suggesting that γδT cells are promising targets in human OSCC with high prognostic values and therapeutic potential.^94^ Yet, the recruited γδT cells appear to be influenced by the tumor microenvironment, which can produce IL‐17A or IFN‐γ depending on the tumor stage.^95^ Nevertheless, the unique properties of γδT cells, such as MHC‐independent anticancer activity, tissue tropism, and reactivity against a broad spectrum of tumors, make them ideal for anticancer therapeutic approaches.

## 8 CONCLUDING REMARKS

While this review indicates that oral LCs and γδT cells are involved in the development of OSCC, our understanding of the mechanisms by which these cells act in the various stages of OSCC is limited and requires further investigation. This is of particular importance during the initiation stage of OSCC, given the role of LCs and γδT cells as steady‐state sentinels of the oral epithelium. Although significant progress has been made in our understanding of the ontogeny of oral LCs, the precise mechanisms by which the oral epithelium regulates the differentiation of local APCs under pathological conditions, particularly during the development of OSCC, remain to be elucidated. The development of oral γδT cells is also beginning to be uncovered, as well as their function in oral immunity. While the role of γδT cells in cancer development has been extensively studied in many malignancies, OSCC has received little attention. Future investigations will provide new insights into the functional repertoire of oral LCs and γδT cells and contribute to the successful translation of these epithelial sentinels into cancer vaccination and immunotherapy.

## Data Availability

Data sharing is not applicable—no new data are generated.
